# Rapid and concise quantification of mycelial growth by microscopic image intensity model and application to mass cultivation of fungi

**DOI:** 10.1038/s41598-021-03512-4

**Published:** 2021-12-17

**Authors:** Soo Kweon Lee, Ju Hun Lee, Hyeong Ryeol Kim, Youngsang Chun, Ja Hyun Lee, Chulhwan Park, Hah Young Yoo, Seung Wook Kim

**Affiliations:** 1 Department of Chemical and Biological Engineering, Korea University, 145, Anam-Ro, Seongbuk-Gu, Seoul, 02841 Republic of Korea; 2 Department of Interdisciplinary Bio-Micro System Technology, College of Engineering, Korea University, 145 Anam-Ro, Seongbuk-Gu, Seoul, 02841 Republic of Korea; 3 Department of Food Science and Engineering, Dongyang Mirae University, 445, Gyeongin-Ro, Guro-Gu, Seoul, Republic of Korea; 4 Department of Chemical Engineering, Kwangwoon University, 20 Kwangwoon-Ro, Nowon-Gu, Seoul, 01897 Republic of Korea; 5 Department of Biotechnology, Sangmyung University, 20, Hongjimun 2-Gil, Jongno-Gu, Seoul, 03016 Republic of Korea

**Keywords:** Biotechnology, Microbiology

## Abstract

The microbial food fermentation industry requires real-time monitoring and accurate quantification of cells. However, filamentous fungi are difficult to quantify as they have complex cell types such as pellet, spores, and dispersed hyphae. In this study, numerous data of microscopic image intensity (MII) were used to develop a simple and accurate quantification method of *Cordyceps* mycelium. The dry cell weight (DCW) of the sample collected during the fermentation was measured. In addition, the intensity values were obtained through the ImageJ program after converting the microscopic images. The prediction model obtained by analyzing the correlation between MII and DCW was evaluated through a simple linear regression method and found to be statistically significant (*R*^2^ = 0.941, *p* < 0.001). In addition, validation with randomly selected samples showed significant accuracy, thus, this model is expected to be used as a valuable tool for predicting and quantifying fungal growth in various industries.

## Introduction

Microorganisms have played an important role as producers in various bio-industries such as food, cosmetics, pharmaceuticals, biomaterials, and fuels^1–4^. In particular, in the bio-industry, microbial fermentation produces not only food, but also a variety of supplements such as antioxidants, flavors, colorants, preservatives, and sweeteners^5,6^. According to the BCC Market Research Report on Fermentation Industry, the global market for bioproducts (petroleum, natural gas, plastics/polymers, composites, pharmaceuticals, chemicals, and power) was estimated at $9.7 trillion in 2020. It will increase at a compounded annual growth rate (CAGR) of 4.8% to reach nearly $12.3 trillion by 2025. In particular, the global market for fermented products (excluding biofuels and biopolymers) is expected to grow at a CAGR of 17.7% over the next five years to reach $69 billion by 2025. The bio-industry growth is due to the rapid development of fundamental life sciences and advanced biotechnology, such as genetic engineering, process engineering, mass production, and purification^7^.

Fermentation is a metabolic process that causes chemical changes in organic substrates through enzymatic actions of microorganisms^8^. During microbial fermentation in a bioreactor, environmental factors such as temperature, dissolved oxygen, pH, agitation rate, and monitoring of cell and nutrient concentrations are very important in mass production^9,10^. In particular, it is well known that the shape and concentration of cells during fermentation can affect the productivity of targeted metabolites^11–15^. Therefore, in fermentation, an understanding of the correlation between the growth of microorganisms and the production of metabolites is required, and various cell quantification techniques have been developed.

**Figure 1:**
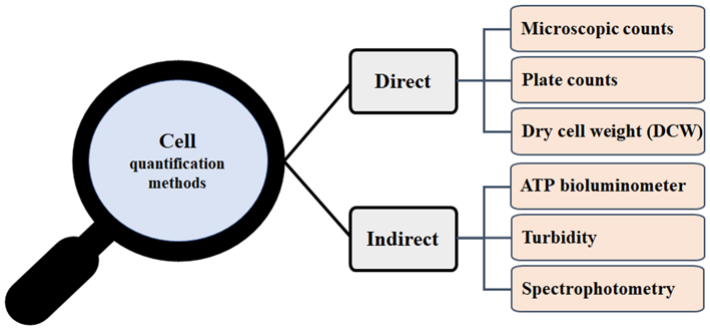
Schematic diagram showing direct and indirect cell quantification methods.

In general, cell quantification is divided into direct and indirect measurements. Figure 1 shows a schematic diagram of cell quantification, including representative examples of direct and indirect techniques. The most well-known direct methods are microscopic cell count, plate medium, and dry cell weight (DCW) measurements. Indirect methods include ATP bioluminescence measurements, turbidity measurements, and spectrophotometric measurements^16,17^.

Among direct methods, the DCW method is useful by measuring the weight of filamentous fungi that do not grow in a certain form^18^. However, before weighing the sample, it must be centrifuged and dried. Therefore, the analysis takes a long time. In addition, real-time monitoring is difficult. On the other hand, the indirect method has a relatively short analysis time, and real-time monitoring is relatively easy. Though, most of the applicable samples are limited to microbes with uniform shapes such as *Escherichia coli*, *Bacillus*, and yeast. It is difficult to apply an indirect method to filamentous fungi or mycelium that grow in the shape of a branch. In addition, if the sample contains non-cellular or colored substances, it may interfere with the measurement and decreases the accuracy of the result^16,19^.

Recently, technologies that overcome deficiencies of direct and indirect methods have been reported. However, most reports have applied fractals to analyze mycelial growth and develop models through correlation with metabolites produced^12,13^. Those models could be used to understand the characteristics of cells growing in complex shapes. However, they are not suitable for quantitative analysis. Therefore, fast and accurate cell quantification techniques applied to the bio-industry for fermenting fungal mycelium are needed.

In this study, a concise image analysis model was designed for quantifying fungal mycelium more quickly and accurately. A microscopic image intensity (MII) model was designed to analyze the correlation between the intensity value of hyphae morphological image and the weight of dry cells. It was based on the linear regression model targeting *Cordyceps militaris*, a filamentous fungus with a non-uniform cell shape. This strain is an improved strain for the production of cordycepin as a functional biomaterial in our previous study^20^. Its optimal production conditions have been determined. Finally, the developed MII model was evaluated by comparing predicted and experimental values of mycelial growth of *C. militaris*.

## Results

### Screening of mycelial growth

In our previous study, *C. militaris* was first employed to produce cordycepin, known as a bioactive substance. As the most effective producer, strain KYL05 was finally selected^20^. Culture conditions and nutrient compositions were determined based on cordycepin production. A medium composition containing 2% glucose and 2% casein hydrolysate was found to be the most effective for its production^20^. In this process, numerous repeated experiments were performed to derive the optimum conditions. The concentration of the final product, cordycepin, was analyzed relatively faster using HPLC^20^. However, growth measurement is a major delay factor in the analysis of fermentation profiling due to the long drying time for the preparation of dry cell weight. Therefore, a rapid quantification technique of cell density is needed for applications such as scale-up and process optimization.

Reported methods are suitable for measuring the density of cells that appear round or oval in shapes, such as bacteria and yeast^21–23^. However, it is difficult to apply the DCW method to the mycelium of fungi that grow in complex shapes. To solve this problem, a new model was suggested and investigated using *C. militaris* KYL05.

### Correlation between microscopic image intensity & DCW

In the initial stage of fermentation process, most cells existed in the form of spores. It was observed that the amount of mycelium rapidly increased at around three days. At this point, cells had grown in the form of spore and elongated hyphae. From the third day, more hyphae began to be observed than spores. On the fourth day, most of the mycelium grew into complex and elongated branches and spores. The shape of this mycelium was maintained up to the sixth day. More mycelium in the form of a pellet rather than a spore began to be observed.

**Figure 2:**
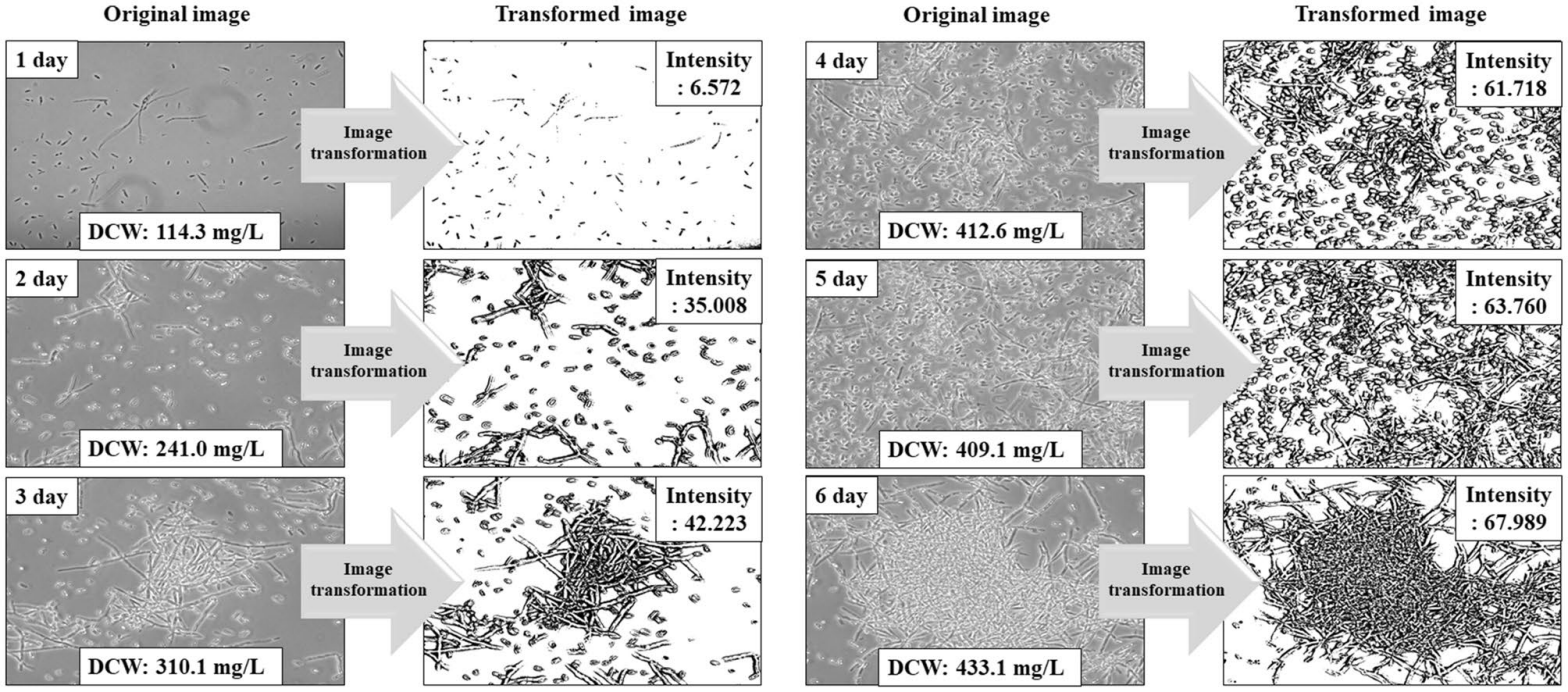
Original microscopic image of mycelial growth according to culture time of *C. militaris* KYL05 and transformed image for measuring intensity with Image J.

**Figure 3:**
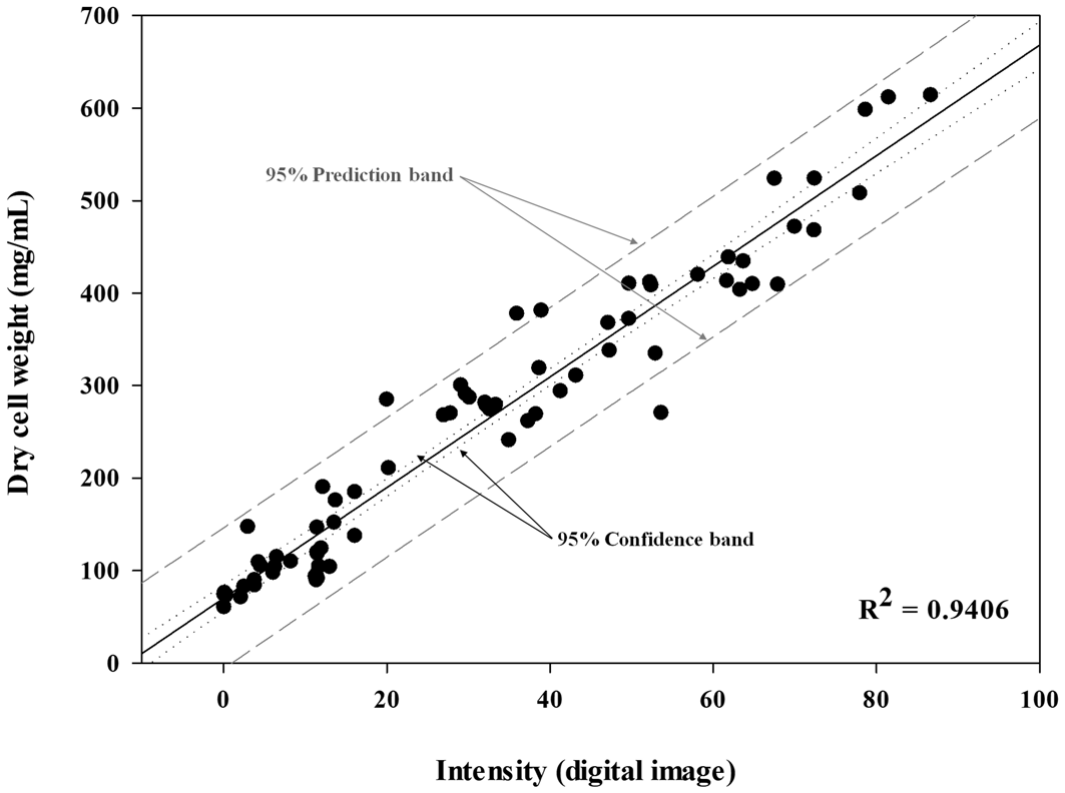
Correlation between microscopic image intensity (MII) of transformed images and dry cell weight (DCW) from quantification of mycelial growth.

Microscopic hyphal images collected over six days were transformed to determine their respective intensity values and used to investigate the relationship between mycelial mass and morphological changes (Fig. 2). From the results of Fig. 2, the hyphae intensity values were correlated with the DCW. During fermentation, DCW was increased, and the intensity of mycelium also increased. The mycelium concentration was gradually increased between the second and third days. The intensity value also increased from 35.01 to 42.22. In addition, at the fourth, fifth, and sixth days, intensity values increased to 61.72, 63.76, and 67.99, respectively. So, the intensity value showed the same pattern as the DCW value, and it could be inferred that there is a correlation between them. DCW and microscopic image intensity values of samples were collected during fermentation. Based on their correlations, the MII model was established (Fig. 3).

### Verification of model

For accurate verification, collected data were analyzed in the SPSS program by simple regression analysis and summarized in Table 1. Analyzed contents showed an equation of Y = 70.095 + 5.982X. According to ANOVA, F value of 1156.825 (*p* < 0.001) was obtained, indicating that the MII model was a suitable model. Also, the coefficient of determination (*R*^2^) was 0.941, showing an explanatory power of 94.1%. This proved that 95% of the variance of actual DCW measurement (a dependent variable) could be explained by the intensity (an independent variable). According to the analysis, the significance probability (*p*) was less than 0.05, confirming that DCW could be measured by intensity.

**Table 1:** MII model verification by SPSS program.

Variable	Unstandardized coefficients		Standardized coefficients	t (p)	F (p)	*R*^2^
	${\beta }_{0}$	Standard error	${\beta }_{1}$			
(Constant)	70.095	7.038	–	9.960***	1156.825***	0.941
MII	5.982	0.176	0.970	34.012***		

*p < 0.05, **p < 0.01, ***p < 0.001.

### Degree of accuracy

**Figure 4:**
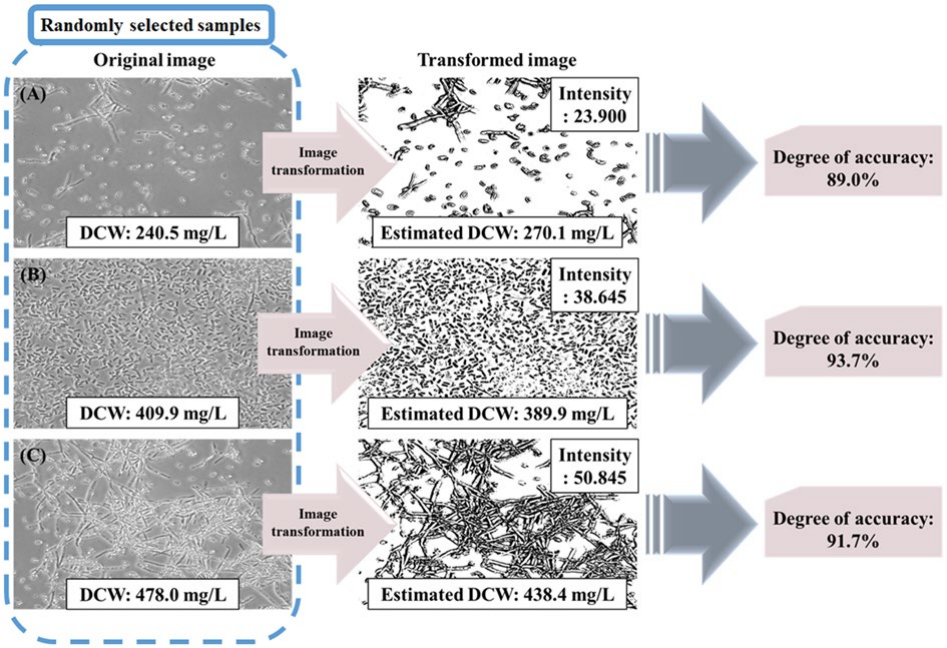
Microscopic images of *C. militaris* KYL05 fermentation were selected from random samples of (**A**, **B**, **C**). The expected DCW was measured from the image intensity value. It was then compared with the real DCW. The degree of accuracy was 89.0% for (**A**), 93.7% for (**B**), and 91.7% for (**C**).

The mycelium intensity and actual DCW values were randomly compared to confirm the model's applicability (Fig. 4). The image intensity value of sample (A) was measured to be 23.9 and the DCW was 270.1 mg/L. The actual DCW value was 240.5 mg/L, confirming an accuracy of 89%. The image intensity of sample (B) was 38.645 and the expected DCW was 383.9 mg/L. The actual DCW value was 409.9 mg/L, showing an accuracy of 93.7%. For sample (C), the intensity value was 50.845 and DCW was 438.4 mg/L, showing an accuracy of 91.7%. These results confirmed that the fungal mycelium could be quantified through the MII model.

### Effect of dilution factors

**Figure 5:**
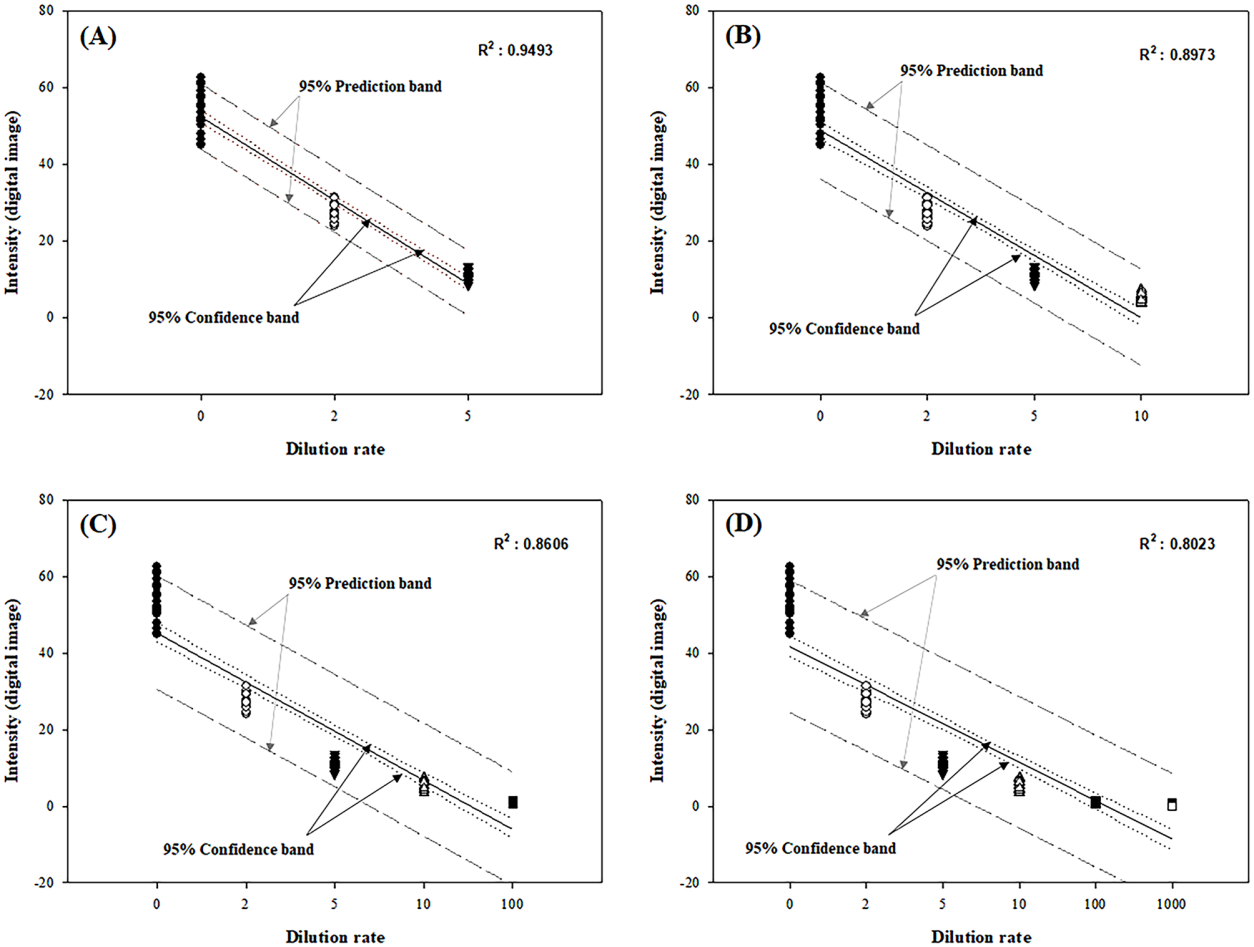
Effect of dilution factors (2, 5, 10, 10^2^, 10^3^) applied to the sample on intensity measurements. The *R*^2^ was 0.9493 for (**A**), 0.8973 for (**B**), 0.8606 for (**C**), and 0.8023 for (**D**).

In addition, to investigate the effect of the dilution factor on the intensity value, various dilution factors (2, 5, 10, 10^2^, and 10^3^) were applied to the *C. militaris* fermented samples (Fig. 5). As a result, it was confirmed that the *R*^2^ values in (B), (C), and (D) decreased from 0.8973 to 0.8606 and 0.8023, respectively. Hence, that culture samples at dilutions of 10, 10^2^, and 10^3^ were not suitable for analysis through the MII model. On the contrary, (A) showed that the *R*^2^ value was measured as high as 0.9493 between dilution factors 2 to 5. Therefore, if a dilution factor of 2 to 5 is applied to the sample, it is expected that the accurate measurement of DCW through the MII model will be possible.

## Discussion

The aim of this study was to design a new model for quantifying fungal mycelium. As a result, we have suggested a model that can measure mycelium immediately and accurately. Likewise, several similar models have been reported in various industries to determine mold shape and quantity^24^. Actually, productivity, which is considered the most important factor from an industrial perspective, is related to mycelium's shape and quantity^24,25^. Therefore, several types of methods have been reported for analyzing fungal mycelium. These image analysis methods were compared in detail with our model, and it was summarized in Table 2.

**Table 2:** Summary of various image analytical methods for fungal growth.

No	Strain	Culture type	Image acquisition	Image transformation & analical method	Application	References
1	*Penicillium decumbens* JU-A10	Solid-state	Digital camera	Matlab, Fractal dimension	On-line determination of fungal growth	Duan et al. (2012)
2	*Rhizopus oligosporus* NRRL-2710	Solid-state	Stereomicroscope	Image J, Fractal dimension	Characterization of fungal growth	Díaz et al. (2010)
3	*Aspergillus fumigatus* PL-12/10	Submerged	Microscopy	Image J, Fractal dimension	Measure the fungal growth	Rajković et al. (2019)
4	*Cephalosporium acremonium* M25	Submerged	Microscopy	Image-Pro, Fractal dimension	Predicted fungal growth & Cephalosporin C (CPC) productions	Kim et al. (2005)
5	*Aspergillus niger* PM1	Submerged	Microscopy	Image J, Fractal dimension	Quantify of fungal growth	Papagianni (2006)
6	*Aspergillus niger* SKAn1015	Submerged	Microscopy	Image J, Fractal dimension	Characterization of fungal	Wucherpfennig et al. (2013)
7	*Penicillium chrysogenum* P-14	Submerged	Flow-cytometry	Fluorescence, Matlab	Fast measurement of fungal growth	Ehgartner et al. (2017)
8	*Penicillium chrysogenum* P-14	Submerged	Flow-cytometry	Fluorescence, Matlab	Estimate of the relationship between fungal morphology, viability, and productivity	Veiter et al. (2019)
9	*Alcaligenes eutrophus* NCIMB 11,599	Submerged	Fluorescence spectroscopy	Matlab	Predicted fungal growth	Hagedorn et al. (2003)
10	*Bacillus polymyxa* POL4-2	Submerged	Fluorescence spectroscopy	Fluorophore	On-line monitoring of fungal growth & antibiotic polymyxin B	Lantz et al. (2006)
11	*Claviceps purpurea* 1029 NS	Submerged	Fluorescence spectroscopy	Fluorophore	Bioprocess monitoring of fungal growth	Boehl et al. (2003)
12	*Saccharomyces cerevisiae* CEN.PK.113-7D	Submerged	Fluorescence spectroscopy	Fluorophore	Estimation of the fungal growth during cultivations	Haack et al. (2004)
13	*Cordyceps militaris* KYL05	Submerged	Microscopy	Image J, IBM SPSS	Immediately measurement of cell mass	This study

In general, fungal fermentation methods can be divided into two types: solid cultivation and submerged cultivation. It is well known that the quantification of fungal mycelium is difficult in a solid medium^26,27^. There are some methods of harvesting colonies and measuring the suspension by spectroscopy to solve this problem. However, their reproducibility and accuracy are low. Recently, several studies have been conducted to improve these problems. Duan et al.^28^ have used *Penicillium decumbens* JU-A10 strain to construct a model to quantify the hyphae matrix's morphological changes in solid fermentation. The amount of fungal mycelium in the solid medium was predicted through the validation of the proposed model. The relative error was 0.54% to 5.22% for biomass and 0.45% to 3.89% for fractal dimension. Matlab and fractal dimensions were used for mycelium image transformation and analysis in that study^28^. Díaz et al.^29^ have also reported the same type of culture condition for characterizing macro and micro structural development of *Rhizopus oligosporus* NRRL-2710 colonies growing on solid media in Petri dishes through image processing and fractal dimension. They found that growth of the colony front was useful for evaluating parameters of fungal development such as the number of tips and the average hypha length^29^.

Other types of methods for microscopic observation of fungal mycelium in submerged cultivation have also been reported. Rajković et al.^30^ have used fractal analysis of microscopic images (FAMI) to measure fractal dimensions (D). Obtained data of D were modeled for the prediction of the growth rate of *Aspergillus fumigatus* PL-12/10^30^. Kim et al.^12^ and Lim et al.^13^ have also investigated the relationship between the morphology and rheological properties of *Cephalosporium acremonium* M25 in a 2.5L bioreactor by fractal dimension based on Cephalosporin C (CPC), a secondary metabolite. Likewise, *Aspergillus niger* PM1 and SKAn1015 have been investigated using ImageJ and fractal dimensions to quantify and characterize mycelium growth^31,32^. These methods and models are useful for the prediction of mycelial form and mycelial development. However, they are not suitable for mycelial quantification.

Filamentous fungi have a wide variety of morphological forms in submerged culture. These could appear as dispersed hyphae, interwoven mycelial aggregates, or denser hyphal aggregates, the so-called pellets^33^. In such cases, flow cytometry (FC) is a useful method for analyzing mycelial aggregates in the form of pellets. FC is a technique used to detect and measure physical and chemical properties of the population of cells or particles. Tens of thousands of cells can be tested quickly. Matlab could be used for data analysis. In fact, this method is suitable for analyzing mycelial aggregates in the form of pellets but not for other types of hyphae^33,34^.

Similarly, fluorescence spectroscopy is a type of electromagnetic spectroscopy that can analyze the fluorescence of a sample. This is a method that employs the fluorescence of a sample by excitation of electrons of a specific compound molecule and emitting light^33,35,36^. According to Boehl et al.^37^, this method is useful for measuring the productivity of mycelium quantity and protein or alkaloid concentration^37^. However, this method could be disturbed by substances other than mycelium during sample analysis, resulting in low accuracy. In addition, it is difficult to measure mycelium that is not in a uniform shape. A method of measuring cell mass by calculating the fluorescence intensity value of *Saccharomyces cerevisiae* using multiple wavelengths has also been reported^38^. However, it was not suitable for mycelium quantification for the same reason.

In this study, an MII model was developed based on the intensity of a microscopic image through simple linear regression analysis. Compared to previously reported methods, it could greatly save time for analyzing the amount of mycelium. The simple regression analysis applied to verify the model can be applied to various fields based on experience and intuition. It can grasp patterns and relationships and convert them into useful information without using experimental data^39,40^. Through this method, the amount of mycelium can be predicted with an accuracy of more than 94%. However, the model's accuracy has only been demonstrated for *C. militaris* KYL05 species. Further studies are needed using other species. Nevertheless, based on these results, we are confident that the MII model will enable hyphae monitoring when applying complex bioprocesses for fungal fermentation. It will provide basic information for controlling large-scale fermentation processes in the future.

In conclusion, a rapid and concise quantification of the *Cordyceps* mycelium was required, and MII based analytical method was applied in this study. The prediction model was derived through the correlation between MII and DCW during fermentation of *C. militaris* KYL05. The MII model was validated by applying a simple linear regression analysis in the SPSS program, as a result, statistical significance (*R*^2^ = 0.941, *p* < 0.001) was confirmed. Therefore, by analyzing the image intensity of the mycelium collected through a microscope, it is possible to rapidly estimate the DCW. In addition, validation using randomly selected samples showed high accuracy, suggesting that the MII model enables rapid analysis of DCW during fungal fermentation in the bio-industry.

## Materials and methods

### Microorganisms

In our previous work, *C. militaris* KCTC6064 was purchased from the Korea collection for type cultures (Jeongeup-si, Jeollabuk-do, Korea)^20^. The wild-type of *C. militaris* KCTC6064 was mutated by ultraviolet irradiation. The *C. militaris* KYL05 strain was then obtained^20^. This strain was used in the present study. Each month, organisms were transferred to potato dextrose agar slants to maintain storage culture.

### Culture conditions of *C. militaris*

The basal seed medium was potato dextrose broth (PDB; composition, 4 g/L potato starch, and 20 g/L glucose). The seed culture was performed in a 250 mL Erlenmeyer flask containing 50 ml of the basal seed medium. Culture was performed at 25 °C with pH 6 for three days in a shaking incubator (200 rpm). The main medium was made with the following ingredients: 20 g/L casein hydrolysate, 20 g/L glucose, 0.1 g/L KH_2_PO4, 0.2 g/L K_2_HPO_4_∙3H_2_O, and 0.2 g/L MgSO_4_·7H_2_O^20,41^. The inoculum (4%, v/v) of seed broth of *C. militaris* KYL05 was transferred into the main medium. The cultivation was performed in a 250 mL Erlenmeyer flask containing 50 ml of broth main medium at 25 °C for six days in a shaking incubator (150 rpm)^11,20,42^.

### Measurement of dry cell weight

Cell growth was monitored every 24 h. After sampling, dry cell weight (DCW) was measured. After cultivation, the cultural broth was centrifuged at 8,000 × g for 30 min at 4 °C. The sediment was then washed with distilled water. DCW was measured by samples weight through a pre-weighed filter paper (Whatman GF/C) and dried in a vacuum oven for 48 h at 60°C^11,20^.

### Image transformation from optical microscope image of *C. militaris*

Images of *C. militaris* KYL05 were captured at 24 h intervals during six days of cultivation using a microscope (Olympus BX51 model, Japan). The color (24 bits) images of the whole colonies obtained through the microscopy were converted to greyscale (8 bits) maps to black and white images using Image J program (version 1.46) (https://imagej.nih.gov/ij/download.html). It was automatically selected for the best range of given the image's intensity values based on the percentage of the total number of pixel values from the lowest to highest pixel value. At 8 bits, the gray level range was 0 to 255. The thresholding process was applied to each image by manually adjusting the level to 154^43^. In the 8-bit image of the border, the mycelia and media from the image of the growing front of the colony were virtually separated using the programs subtract background tool (digital filter). Contrast was then enhanced, followed by thresholding to 180 in the gray-scale and dilated using the dilate tool. The identification of pixels not belonging to the mycelium was done using media filters and tools to find the maximum. In order to remove noise from the original optical microscope image, a transformed image was obtained by removing pixels not belonging to the mycelium. Intensity from each transformed image was measured with the ImageJ program^19,43^.

### Simple linear regression model between the intensity of transformed image and DCW

The IBM Statistical Package for the Social Sciences (SPSS) Statistical 27.0.0 program (https://www.ibm.com/kr-ko/analytics/spss-statistics-software) was used to evaluate variables of image intensity. Among several statistical methods, a simple linear regression model that could analyze the relationship with the dependent variable by considering only one independent variable was used with the following Eq. (1):

1 $${\text{y}} = \beta_{0} + \beta_{1} x + \epsilon$$

where y was the predicted value of the dependent variable (y) for any given value of the independent variable (x); ${\beta }_{0}$ was the intercept, the predicted value of y when the x was 0; ${\beta }_{1}$ was the regression coefficient (expect y to change as x increases); *x* was the independent variable (the variable expected to influence y); and $\in$ value was the error of the estimate, or the variation in our estimate of the regression coefficient^44,45^.

Through this, the mean, standard deviation, and residual variables were analyzed, along with the 95% confidence interval. In addition, the hypothesis was tested through the analysis of variance (ANOVA). The significance level was at *p* < 0.05.

## Data Availability

The datasets generated and analyzed during the current study are available from the corresponding author on reasonable request.
